# Mutations in the influenza virus, primarily H5N1, enhance the virus’s virulence, favor receptor interaction, and increase drug resistance

**DOI:** 10.1097/JS9.0000000000002403

**Published:** 2025-04-22

**Authors:** Chiranjib Chakraborty, Arpita Das, Manojit Bhattacharya, Md. Aminul Islam

**Affiliations:** a Department of Biotechnology, School of Life Science and Biotechnology, Adamas University, Kolkata, West Bengal, India; b Department of Zoology, Fakir Mohan University, Vyasa Vihar, Balasore, Odisha, India; c COVID-19 Diagnostic Lab, Department of Microbiology, Noakhali Science and Technology University, Noakhali, Bangladesh; d Advanced Molecular Lab, Department of Microbiology, President Abdul Hamid Medical College, Karimganj, Kishoreganj, Bangladesh

The recent emergence of the highly pathogenic avian influenza (HPAI) H5N1 virus has created a health emergency in the USA. In early 2024, HPAI H5N1 strain A was detected in Texas, and it was noted as a human-infecting bovine virus (A/Texas/37/2024, Texas). The USA performed a genetic analysis of influenza’s hemagglutinin (HA). The analysis indicated that this virus (A/Texas/37/2024, Texas) was associated with H5 clade 2.3.4.4b^[^1^]^. Infection was spread to dairy cows in the USA. The virus infected more than 875 cows^[^2^]^. The USA CDC indicated that, with the 2.3.4.4b clade of the virus, at least 15 humans were infected worldwide. On the other hand, the USA CDC reported that 17 humans have been infected in the USA with H5N1 with the 2.3.4.4b clade since 2022^[^1^]^. However, researchers have indicated that several mutations of H5N1 have been discovered from time to time, which might control virulence properties in receptor interactions with the virus. The mutations might enhance the virus’s virulence properties and create an environment for favorable receptor interactions.

Recently, two mutations, E186D and Q222H, were reported in the hemagglutinin (HA) gene of the H5 subtype, which Favors receptor interaction. Similarly, one mutation (E627K) was reported in the polymerase basic 2 (PB2) gene, which enhances the virulence properties of the virus. A critical illness was noted in the H5N1 infection of a 13-year-old Canadian girl in the adolescent period. She was suffering from obesity and mild asthma and had a fever. Finally, there was a progression of the heart failure of the girl. She was positive for the RT-PCR test specific for influenza A(H5) and was detected as positive. The girl was treated with oseltamivir, baloxavir, and amantadine and recovered. The specimens from upper-respiratory specimens were collected, and they showed consistently higher Ct values, indicating a reduction in the viral RNA load in serum. The virus genome sequence shows several mutations. The E627K mutation was detected in PB2 with 52% allele frequency. Similarly, two mutations were detected in the HA gene, which is E186D with 28% allele frequency and Q222H with 35% allele frequency^[^3^]^ (Table 1).

**Table 1:** Significant mutations of the influenza virus, especially H5N1 type A.

Sl. No.	Mutation	Gene name	Remarks	Reference
1.	E627K	PB2	This mutation possibly related with the enhanced virulence	^[^2^]^
2.	E186D	HA	This mutation support to binding of receptors interaction	
3.	Q222H	HA	It enhanced receptor binding interaction	^[^2^]^
4.	Q226L	HA	It increased receptor binding interaction	^[^1^]^
5.	T199I	HA	This mutation support to increased binding of receptors interaction	^[^7^]^
6.	T192I	HA	Associated with enhanced receptor binding specificity through the augmented α2,6-SA	^[^8^]^
			(Salicylic acid) recognition	
7.	S137A	HA	Associated with enhanced receptor binding specificity through the augmented α2,6-SA	^[^8^]^
			(Salicylic acid) recognition	
8.	H275Y	NA	It linked with resistance to the antiviral drug oseltamivir	^[^9^]^
9.	I222V	NA	Associated with resistance to the antiviral drug oseltamivir	^[^10^]^
10.	S31N	M2	Associated with confer amantadine resistance	^[^10^]^

In the mutation E627K in PB2, an alteration from glutamic acid to lysine was noted at position 627, which was associated with increased virulence. Previously, Jonges *et al* reported that the PB2 E627K mutation in HAPI H7N7 type A is responsible for fatal human case^[^4^]^. Along with the group, the researchers have also reported that this specific mutation in avian influenza viruses dramatically increases the public health risk, and the virus has pandemic potential^[^4,5^]^. The mutations HA gene increase binding affinity to sialic acids (α2,3- and α2,6-linked sialic acids), which act as receptors for viral entry into cells. Recently, Dadonaite *et al* reported that HA gene (H5) mutations augment binding affinity to sialic acids (α2,6-linked) and act as receptors that facilitate viral entry into human respiratory tract cells. Finally, the cells facilitate viral replication^[^6^]^.

Recently, the other four mutations in the noted HA gene are Q226L, T199I, S137A, and T192I. These mutations help increase receptor binding activity and, thereby, help enter the host. Lin *et al* reported that Q226L substitution in HA helps change human receptors’ specificity. At the same time, another mutation, N224K, will enhance the specificity of human receptors along with the Q226L. Similarly, Good *et al* illustrated that T199I mutation in HA is accountable for increased binding breadth^[^7^]^. Conversely, Yang *et al* demonstrated that two mutations, S137A and T192I, in HA, are associated with enhanced receptor binding specificity through the augmented α2,6-SA (Salicylic acid) recognition^[^8^]^.

Again, mutations such as H275Y and N71S in the NA (Neuraminidase) gene are responsible for antiviral drug resistance. Farrukee *et al* reported that H275Y in NA substitution confers oseltamivir resistance^[^9^]^. Similarly, Baz *et al* reported that I222V in NA is responsible for oseltamivir resistance. Likewise, Baz *et al* reported that S31N in NA confers for oseltamivir resistance^[^10^]^ (Fig. 1).

**Figure 1.**
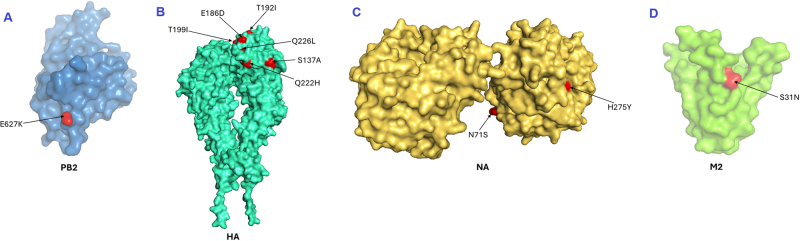
Location of different significant mutations of H1N5 in polymerase basic 2 (PB2) hemagglutinin (HA), neuraminidase (NA) and M2. (A) Significant mutation in PB2. (B) Significant mutations in HA. (C) Significant mutations in NA. (D) Significant mutation in M2.

GISAID is a global resource that provides access to influenza virus data (especially genomic data)^[^11^]^. GISAID is one of the significant influenza virus databases. All Influenza Data from GISAID indicated several mutations in different segments.

The data shows the significant mutations of PB2 segments, which are D701N (A/dairy_cow/USA/004223-001/2025|2025); D701N (A/ring-billed_gull/USA/004743- 01/2025|2025); E627K (A/bear/USA/008592-001/2025|2025); E627K (A/bear/USA/008597-004/2025|2025), E627K (A/Wyoming/01/2025| 2025-02-11), E627X (A/British_Columbia/PHL-2032/2024|2024-11), E627K (A/dairy_cow/USA/004631-001/2025|2025), and E627K (A/bottlenose_dolphin/Florida/24-038155-004/2023 | 2023-12-31) (Fig. 2A).

**Figure 2.**
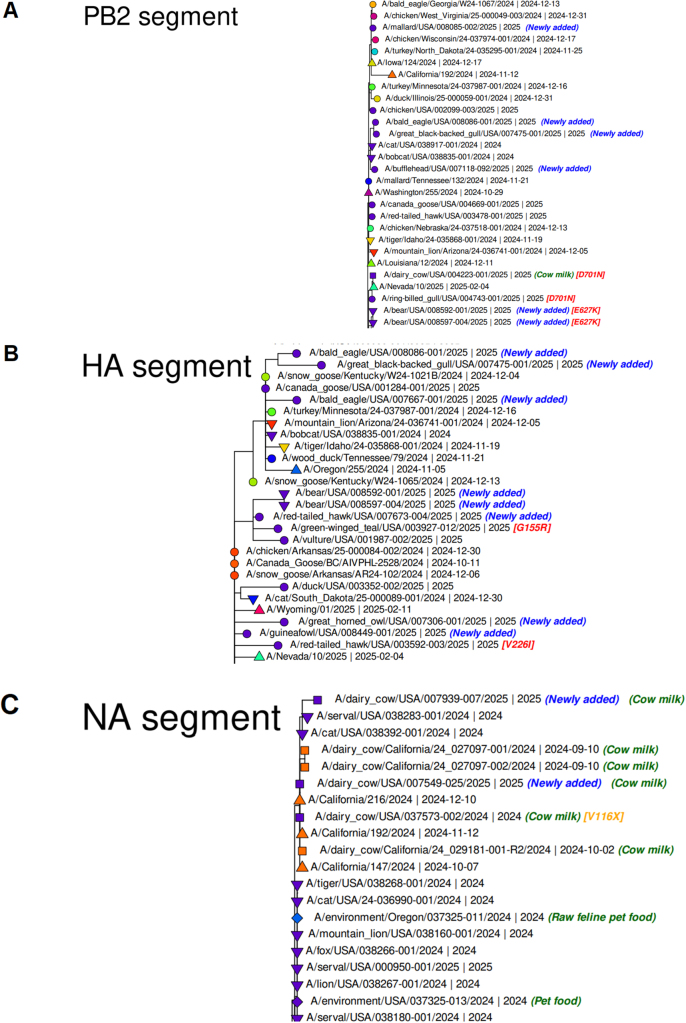
GISAID reported different significant recent mutations in different segments of H1N5. GISAID has depicted a phylogenetic tree, and mutation is reported in front of the branch of a phylogenetic tree. (A) Significant mutations of PB2 represented through the phylogenetic tree. (B) Significant mutations of HA represented through the phylogenetic tree. (C) Significant mutations of NA represented through the phylogenetic tree. This figure has been taken from GISAID.

Similarly, the data demonstrates the significant mutations of HA segments which are G155R(A/green-winged_teal/USA/003927-012/2025),V226I(A/red-tailed_hawk/USA/003592-003/2025 | 2025), E202X, Q238X (A/British_Columbia/PHL-2032/2024| 2024-11), D171N (A/red-tailed_hawk/USA/003573-002/2025|2025),V226I(A/chicken/Oregon/24-035148 01/2024|2024-11-18),S110N (A/dairy_cow/USA/002093-001/2025 | 2025) (Fig. 2B).

The data shows the significant mutations of NA segments that are V116X (A/dairy_cow/USA/037573-002/2024 | 2024); S248N(A/bear/USA/008597-004/2025 | 2025); S248N (A/bear/USA/008592-001/2025 | 2025), S248N (A/red-tailed_hawk/USA/007673-004/2025 | 2025), S247N (A/Washington/253/2024 | 2024-10-24); S248N and V116A (A/canada_goose/USA/003297-006/2025 | 2025); S247N(A/Washington/253/2024 | 2024-10-24), etc. (Fig. 2C).

Recent infections of H5N1 have been transmitted from dairy cows to farm workers in the USA and also started to spread from dairy cows to farm workers in other parts of the world, such as Egypt^[^12^]^. At the same time, H5N1 mutates quickly, similar to other RNA viruses like SARS-CoV-2^[^13^]^. Recent infections of H5N1 are transmitted from dairy cows to farm workers. Recent mutations are considered a significant risk factor for humans, so the virus can emerge as a new pandemic strain. The mutations in H5N1 with pandemic potential are worrisome, so continuous monitoring is needed for the currently circulating H5N1 virus.

## Data Availability

No datasets generated during and/or analyzed during the current study.
